# Development of a novel nomogram to predict hemorrhagic transformation following endovascular treatment in patients with acute ischemic stroke

**DOI:** 10.3389/fneur.2025.1564063

**Published:** 2025-07-08

**Authors:** Xiaofen Zhao, Yuanjie Le, Ting Xin, Guosheng Gao, Mengya Zhu, Kai Xun, Xinliang Mao

**Affiliations:** ^1^Department of Intensive Care Medicine, Ningbo No. 2 Hospital, Ningbo, China; ^2^Department of Emergency Medicine, Ningbo No. 2 Hospital, Ningbo, China; ^3^Department of Neurology, Tai’an Central Hospital, Tai’an, China; ^4^Department of Clinical Laboratory, Ningbo No. 2 Hospital, Ningbo, China; ^5^Department of Rheumatology and Immunology, Ningbo No. 2 Hospital, Ningbo, China

**Keywords:** acute ischemic stroke, endovascular therapy, hemorrhagic transformation, nomogram, prediction factors

## Abstract

**Background:**

Hemorrhagic transformation (HT) is a critical complication of endovascular therapy (EVT) in acute ischemic stroke (AIS), significantly worsening patient outcomes. Although various risk factors have been identified, existing predictive models often fail to account for the multimodal nature of EVT and the complex interplay of clinical, imaging, and laboratory variables.

**Objective:**

This study aimed to develop and validate a nomogram-based predictive model to estimate the risk of HT in AIS patients undergoing EVT, incorporating clinical, imaging, and laboratory data to provide a comprehensive risk assessment.

**Methods:**

A retrospective analysis was performed on 154 AIS patients who underwent EVT at a single center between 2018 and 2023. The least absolute shrinkage and selection and operator (LASSO) and multivariate logistic regression were used to identify the independent predictors of HT. A nomogram was constructed and evaluated using the area under the receiver operating characteristic curve (AUC-ROC), calibration curves, and decision curve analysis (DCA).

**Results:**

Among the 154 patients, 34.4% experienced HT. The nomogram demonstrated excellent discriminatory ability, with an AUC-ROC of 0.82 (95% CI: 0.752–0.888), and strong calibration, as indicated by calibration curves. DCA confirmed the model’s clinical utility when the threshold probability was <0.8. Six independent prediction factors of HT were identified: atrial fibrillation (OR: 6.152), albumin (OR: 1.145), baseline NIHSS score (OR: 1.081), diastolic blood pressure (OR: 1.057), Trial of ORG 10172 in Acute Stroke Treatment (TOAST) Classification (TOAST_2, cardioembolic stroke subtype, OR: 0.201), and the location of obstructed blood vessel_5 (basilar artery occlusion, OR: 0.081).

**Conclusion:**

The developed nomogram provides an accurate, individualized risk assessment of HT in AIS patients undergoing EVT. This tool enables personalized risk stratification, aiding clinicians in optimizing treatment strategies and improving patient outcomes. Further multicenter validation is warranted to generalize these findings.

## 1 Introduction

Acute ischemic stroke (AIS) is a major contributor to global morbidity and mortality, with its incidence steadily rising due to aging populations and lifestyle changes. AIS accounts for approximately 87% of all strokes, making it the most prevalent stroke subtype and a leading cause of death and long-term disability worldwide (1, 2). The advancement of endovascular therapy (EVT) has revolutionized the treatment of AIS, particularly for cases caused by large vessel occlusion (3). Key EVT modalities include mechanical thrombectomy (MT), balloon angioplasty with stenting, and hybrid approaches that combine MT with other interventions (4). These therapies have demonstrated significant efficacy in improving cerebral perfusion, reducing infarct size, and enhancing functional outcomes (5).

However, a critical complication of EVT is hemorrhagic transformation (HT), which significantly worsens neurological outcomes and increases mortality and disability rates (6). Reported HT incidence ranges from 3 to 49.5%, influenced by definition used in different studies, patient characteristics, treatment modalities, and procedural factors (7, 8). HT not only complicates post-stroke recovery but also poses significant challenges to clinicians in optimizing treatment strategies. Thus, accurate prediction of HT risk across different EVT modalities is essential for guiding patient management, improving outcomes, and minimizing complications (9).

Several studies have identified potential risk factors for HT, including stroke severity, blood pressure, hyperglycemia, coagulation abnormalities, and inflammatory markers (10, 11). Imaging-based parameters, such as ASPECTS scores and early ischemic changes, have also shown predictive value (12). Despite these advancements, current predictive approaches have notable limitations. Most studies focus on a single treatment modality, such as MT, without accounting for differences in HT risk associated with other EVT strategies, including balloon angioplasty and stenting. This limits their applicability to real-world clinical scenarios, where treatment is often multimodal and patient-specific (13).

Moreover, discrepancies in findings across studies highlight the need for a standardized and integrated approach to HT risk prediction. For example, while some studies have associated elevated inflammation factor levels with increased HT risk, their significance across different EVT modalities remains uncertain (14). Existing predictive models are also constrained by their reliance on a limited number of variables, which may overlook the complex interplay of clinical, imaging, and laboratory factors contributing to HT (15).

To address these gaps, this study aims to develop a novel nomogram-based predictive model for assessing HT risk in AIS patients undergoing EVT. By incorporating clinical, imaging, and laboratory data, this study provides a comprehensive analysis of HT risk factors across multiple EVT modalities. The use of advanced statistical methods, such as least absolute shrinkage and selection operator (LASSO) regression and multivariate logistic regression, ensures the selection of robust and independent predictors while minimizing model complexity.

The primary objective of this study is to construct and validate a nomogram that predicts HT risk following EVT. The model evaluates independent predictors specific to various EVT modalities and assesses its predictive performance using metrics such as the area under the receiver operating characteristic curve (AUC-ROC), calibration curves, and decision curve analysis (DCA). By facilitating personalized risk stratification, this study aims to guide clinicians in selecting appropriate treatment strategies, thereby improving patient outcomes and addressing a critical gap in stroke care.

## 2 Methods

### 2.1 Data source

This study retrospectively analyzed data from all consecutive acute ischemic stroke (AIS) patients treated at Ningbo No. 2 Hospital between 2018 and 2023. Patients who underwent MT for large vessel occlusion but were not candidates for thrombolytic therapy were included. Both preoperative and postoperative data were collected, including demographic information, medical history, imaging evaluations, and laboratory findings. Postoperative HT was determined based on imaging results (CT or MRI) obtained within 24 h after the procedure. The Alberta Stroke Program Early CT Score (ASPECTS) was recorded for all patients as a measure of baseline ischemic injury. However, no specific ASPECTS threshold was used as an exclusion criterion for this study. Furthermore, during the study period, a rigid ASPECTS cut-off was not uniformly applied to determine clinical eligibility for EVT at the study center; rather, EVT candidacy was determined based on a comprehensive evaluation of individual patient profiles, including clinical status and overall imaging findings, in accordance with prevailing stroke treatment guidelines.

### 2.2 Inclusion criteria

Patients were included if they were aged 18 years or older, had a confirmed diagnosis of AIS, and were treated for large vessel occlusion using MT, with clearly identified occlusion sites and TOAST classification. Only those who achieved successful recanalization of the target vessel (TICI ≥ 2b) were eligible. Additionally, included patients were required to have complete preoperative data, including medical history, recanalization time window, laboratory indicators, and imaging evaluations, as well as postoperative CT or MRI performed within 24 h to confirm the occurrence of HT.

### 2.3 Exclusion criteria

Patients were excluded if they failed to achieve vascular recanalization (TICI < 2b), had significant intracranial hemorrhage prior to the procedure, lacked key preoperative data such as coagulation or inflammatory markers, or had severe pre-existing organ failure, including end-stage liver or renal failure, or coagulation disorders.

### 2.4 Data collection

#### 2.4.1 Dependent variable

The primary outcome (dependent variable) for the development of this nomogram was the occurrence of any radiologically confirmed HT within 24 h postoperatively, detected via CT or MRI imaging. While HT was sub-classified according to the European Cooperative Acute Stroke Study (ECASS) criteria (e.g., HI1, HI2, PH1, PH2) (16), all subtypes were combined as a single binary endpoint (presence or absence of any HT) for this analysis to ensure statistical power. The systematic evaluation of predictors for specific HT subtypes or for symptomatic HT (sHT, defined as radiological HT with concomitant neurological deterioration) was not the primary focus of this particular nomogram development but remains an objective for future research.

#### 2.4.2 Independent variables

A wide range of potential predictors (independent variables) were included in this study, covering patient characteristics, imaging scores, interventional methods, Time-related variables, and laboratory findings.

Patient characteristics encompassed demographics such as sex and age, as well as comorbidities, including hypertension, diabetes mellitus (DM), hyperlipidemia, atrial fibrillation (AF), and a history of prior stroke. Lifestyle factors such as smoking and alcohol use were also considered, along with medication history, specifically the prior use of antiplatelet or anticoagulant drugs.

Imaging and clinical scores included the National Institutes of Health Stroke Scale (NIHSS) to assess stroke severity and the ASPECTS to evaluate baseline ischemic injury. Stroke etiology was determined using the TOAST classification, and the site of vessel occlusion was also recorded. According to the Trial of ORG 10172 in Acute Stroke Treatment (TOAST) classification system, ischemic stroke can be categorized into five subtypes: large artery atherosclerosis (TOAST_1), cardioembolism (TOAST_2), small artery occlusion (TOAST_3), other specific causes (TOAST_4), and undetermined cause (TOAST_5). The variable “obstructed blood vessel” was classified into five categories: internal carotid artery (ICA, obstructed blood vessel_1), anterior cerebral artery (ACA, obstructed blood vessel_2), middle cerebral artery (MCA, obstructed blood vessel_3), vertebral artery (VA, obstructed blood vessel_4), and basilar artery (BA, obstructed blood vessel_5).

Regarding interventional treatment methods, three approaches were analyzed: MT (1), balloon angioplasty combined with stenting (2), and hybrid treatments involving MT with balloon angioplasty and stenting (3). The second treatment type did not include patients with isolated carotid occlusion without intracranial occlusion. For patients undergoing intracranial stent placement, perioperative antiplatelet and anticoagulant therapy was initiated according to contemporary guidelines and institutional protocols.

Time-related variables included the symptom-to-puncture time (Time_1, measured in hours) and the symptom-to-recanalization time (Time_2, also measured in hours).

Laboratory findings covered a broad set of markers. Coagulation markers included D-dimer (DD), fibrinogen (FIB), prothrombin time (PT), activated partial thromboplastin time (APTT), and thrombin time (TT). Inflammatory markers such as interleukin-6 (IL-6), IL-10, and tumor necrosis factor-alpha (TNF-α) were also evaluated. Hematologic parameters included platelet count (PLT), white blood cell count (WBC), and the neutrophil-to-lymphocyte ratio (NLR). Metabolic markers, including blood glucose (GLU), total cholesterol (TCHO), and LDL/HDL levels, were assessed. Lastly, liver and kidney function markers, such as C-reactive protein (CRP), creatinine (CR), aspartate aminotransferase (AST), alanine aminotransferase (ALT), and albumin (ALB), were included in the analysis.

### 2.5 Statistical analysis

#### 2.5.1 Descriptive analysis

Categorical variables were reported as counts (%) and compared between groups using the chi-square test or Fisher’s exact test. Continuous variables were tested for normality using the Shapiro–Wilk test. Normally distributed data were expressed as mean ± standard deviation (SD) and compared using the independent t-test, while non-normally distributed data were expressed as median (P25, P75) and compared using the Mann–Whitney *U*-test. A *p*-value < 0.05 was considered statistically significant.

#### 2.5.2 Variable selection

Univariate analysis was performed to identify variables potentially associated with HT. Variables with a *p*-value < 0.10 in the univariate analysis were included in a least absolute shrinkage and selection operator (LASSO) regression model to reduce dimensionality and prevent overfitting. Two rounds of LASSO regression were used to identify the most robust predictors of HT. The final set of variables was included in a multivariate logistic regression analysis to identify independent risk factors for HT.

#### 2.5.3 Nomogram construction and validation

A nomogram was constructed based on the independent predictors identified in the multivariate logistic regression analysis. Each predictor’s regression coefficient and odds ratio (OR) were used to determine its weight in the model.

#### 2.5.4 Model performance evaluation

The predictive performance of the developed nomogram was assessed through internal validation using the development cohort. This evaluation focused on discrimination, calibration, and clinical utility. Discrimination was assessed by calculating the area under the receiver operating characteristic curve (AUC-ROC) to measure the model’s ability to differentiate between patients with and without HT. Calibration was evaluated using calibration curves to compare predicted probabilities with observed outcomes, along with the Hosmer-Lemeshow goodness-of-fit test to assess the model’s calibration accuracy. Clinical utility was examined through decision curve analysis (DCA), which determined the net clinical benefit of the nomogram across a range of threshold probabilities.

All statistical analyses were conducted using R version 4.0.3 (R Foundation for Statistical Computing, Vienna, Austria) and SPSS version 26.0 (IBM Corp., Armonk, NY).

## 3 Results

### 3.1 Baseline characteristics of the study population

During the study period from 2018 to 2023, a total of 223 patients who underwent endovascular therapy (EVT) for acute ischemic stroke at the study center were initially assessed for eligibility. Of these, 69 patients were excluded based on the predefined criteria: 38 patients did not achieve successful vascular recanalization (TICI < 2b); 12 patients had significant intracranial hemorrhage prior to the procedure; 8 patients lacked key preoperative data, such as coagulation or inflammatory markers; and 11 patients had severe pre-existing organ failure or other specified exclusion conditions. After applying these exclusion criteria, 154 patients fulfilled all inclusion criteria and constituted the final study cohort.

Among the patients, 53 (34.4%) experienced HT postoperatively, while 101 (65.6%) did not. The baseline demographic, clinical, and laboratory characteristics of patients, stratified by HT status, are summarized in Table 1.

**Table 1:** Baseline characteristics of acute ischemic stroke (AIS) patients undergoing endovascular therapy (EVT).

	Non-HT	HT	Total	Test methods	*p*_value
Number (%)	101 (65.58)	53 (34.42)	154 (100%)		
Interventional treatment methods				Chi-square	0.663
1	84 (83.17)	41 (77.36)	125 (81.17)		
2	5 (4.95)	4 (7.55)	9 (5.84)		
3	12 (11.88)	8 (15.09)	20 (12.99)		
Sex				Chi-square	0.923
Male	37 (36.63)	19 (35.85)	56 (36.36)		
Female	64 (63.37)	34 (64.15)	98 (63.64)		
DM				Chi-square	0.015
No	87 (86.14)	37 (69.81)	124 (80.52)		
Yes	14 (13.86)	16 (30.19)	30 (19.48)		
Hyperlipd				Fisher’s exact test	1
No	98 (97.03)	52 (98.11)	150 (97.40)		
Yes	3 (2.97)	1 (1.89)	4 (2.60)		
Atrial fibrillation				Chi-square	0.003
No	65 (64.36)	21 (39.62)	86 (55.84)		
Yes	36 (35.64)	32 (60.38)	68 (44.16)		
CI history				Fisher’s exact test	1
No	96 (95.05)	50 (94.34)	146 (94.80)		
Yes	5 (4.95)	3 (5.66)	8 (5.20)		
Related drug history				Chi-square	0.123
No	87 (86.14)	50 (94.34)	137 (88.96)		
Yes	14 (13.86)	3 (5.66)	17 (11.040)		
Smoking				Fisher’s exact test	0.552
No	98 (97.03)	53 (100)	151 (98.052)		
Yes	3 (2.97)	0 (0)	3 (1.948)		
Alcohol				Fisher’s exact test	1
No	98 (97.03)	51 (96.23)	149 (96.75)		
Yes	3 (2.97)	2 (3.77)	5 (3.25)		
mRS				Fisher’s exact test	0.629
0	7 (6.931)	2 (3.77)	9 (5.84)		
1	2 (1.98)	1 (1.89)	3 (1.95)		
2	2 (1.98)	0 (0)	2 (1.30)		
3	3 (2.97)	3 (5.66)	6 (3.90)		
4	64 (63.37)	30 (56.60)	94 (61.040)		
5	23 (22.77)	17 (32.08)	40 (25.97)		
TOAST				Fisher’s exact test	0.042
1	37 (36.63)	28 (52.83)	65 (42.21)		
2	53 (52.48)	19 (35.85)	72 (46.75)		
4	5 (4.95)	0 (0)	5 (3.25)		
5	6 (5.94)	6 (11.32)	12 (7.79)		
Obstructed blood vessel				Fisher’s exact test	0.010
1	31 (30.69)	30 (56.60)	61 (39.61)		
2	2 (1.98)	0 (0)	2 (1.30)		
3	51 (50.50)	21 (39.62)	72 (46.75)		
4	5 (4.95)	0 (0)	5 (3.25)		
5	12 (11.88)	2 (3.77)	14 (9.09)		
Age, median (P25, P75)	72 (64, 77)	69 (59, 76)	71 (62, 77)	Mann–Whitney *U* test	0.298
SBP, median (P25, P75)	130 (126, 140)	140 (130, 150)	130 (130, 145)	Mann–Whitney *U* test	0.003
DBP, median (P25, P75)	70 (70, 80)	80 (70, 83)	70.5 (70, 80)	Mann–Whitney *U* test	<0.001
NIHSS, mean (SD)	18.77 (6.79)	21.76 (5.78)	19.80 (6.60)	*t*-test	0.007
ASPECTS, median (P25, P75)	10 (8, 10)	9 (8, 10)	10 (8, 10)	Mann–Whitney *U* test	0.295
Time 1, median (P25, P75)	4.62 (3.08, 6.17)	4.50 (3.37, 6.00)	4.58 (3.17, 6.08)	Mann–Whitney *U* test	0.870
Time 2, median (P25, P75)	5.75 (4, 8)	5.87 (4.5, 8)	5.81 (4.27, 8)	Mann–Whitney *U* test	0.596
DD, median (P25, P75)	503 (208, 1,693)	511 (254, 905)	507 (230.75, 1375.5)	Mann–Whitney *U* test	0.712
PT, median (P25, P75)	11.3 (11.30, 12.10)	11.4 (11.10, 12.40)	11.3 (11.30, 12.18)	Mann–Whitney *U* test	0.806
FIB, median (P25, P75)	413 (351, 492)	395 (338, 478)	404 (347, 486)	Mann–Whitney *U* test	0.328
TT, median (P25, P75)	24 (21.50, 28.20)	24.2 (22.50, 25.60)	24.15 (21.78, 27.83)	Mann–Whitney *U* test	0.566
APTT, median (P25, P75)	29.8 (27.50, 31.60)	29.8 (27.40, 33.50)	29.8 (27.43, 31.88)	Mann–Whitney *U* test	0.270
PLT, median (P25, P75)	171 (142, 219)	194 (145, 232)	178 (142.25, 228.5)	Mann–Whitney *U* test	0.205
WBC, median (P25, P75)	8 (6.30, 9.70)	8.7 (5.90, 11.30)	8.35 (6.20, 10.30)	Mann–Whitney *U* test	0.416
NEU Percent, median (P25, P75)	0.821 (0.75, 0.88)	0.832 (0.77, 0.89)	0.825 (0.76, 0.88)	Mann–Whitney *U* test	0.381
LYM Percent, median (P25, P75)	0.12 (0.07, 0.17)	0.113 (0.07, 0.16)	0.12 (0.07, 0.16)	Mann–Whitney *U* test	0.488
MONO Percent, median (P25, P75)	0.052 (0.04, 0.07)	0.048 (0.03, 0.06)	0.05 (0.03, 0.07)	Mann–Whitney *U* test	0.158
ESO Percent, median (P25, P75)	0.003 (0, 0.009)	0.002 (0, 0.006)	0.002 (0, 0.007)	Mann–Whitney *U* test	0.142
BASO Percent, median (P25, P75)	0.002 (0.002, 0.003)	0.002 (0.001, 0.003)	0.002 (0.001, 0.003)	Mann–Whitney *U* test	0.306
RBC, mean (SD)	3.968 (0.57)	4.235 (0.61)	4.06 (0.60)	*t*-test	0.008
HGB, mean (SD)	123.08 (16.55)	128.17 (21.44)	124.83 (18.47)	*t*-test	0.104
MCV, median (P25, P75)	93.1 (89.70, 96.90)	92.6 (89.60, 94.50)	92.65 (89.70, 96.650)	Mann–Whitney *U* test	0.239
TCHO, mean (SD)	3.747 (0.90)	3.725 (0.890)	3.739 (0.90)	*t*-test	0.890
TG, median (P25, P75)	0.97 (0.61, 1.41)	0.93 (0.60, 1.39)	0.955 (0.60, 1.41)	Mann–Whitney *U* test	0.700
HDL, median (P25, P75)	1.02 (0.84, 1.20)	1.05 (0.90, 1.23)	1.03 (0.84, 1.21)	Mann–Whitney *U* test	0.430
LDL, mean (SD)	2.21 (0.68)	2.14 (0.72)	2.18 (0.70)	*t*-test	0.562
CRP, median (P25, P75)	2.53 (1.19, 6.11)	2.07 (0.94, 6.08)	2.42 (1.11, 6.10)	Mann–Whitney *U* test	0.338
CR, median (P25, P75)	64 (52.10, 75.30)	64.7 (50.60, 82.40)	64.35 (51.93, 77.50)	Mann–Whitney *U* test	0.715
GLU, median (P25, P75)	7.02 (6.14, 8.07)	7.88 (6.65, 9.29)	7.275 (6.335, 8.60)	Mann–Whitney *U* test	0.015
TBIL, median (P25, P75)	12.20 (9.60, 17.10)	14.20 (10.70, 16.50)	13 (10.13, 17.08)	Mann–Whitney *U* test	0.155
DBIL, median (P25, P75)	4.30 (3.40, 5.70)	4.70 (3.90, 6.40)	4.55 (3.53, 6.18)	Mann–Whitney *U* test	0.136
TP, mean (SD)	59.64 (8.511)	61.074 (9.169)	60.133 (8.74)	*t*-test	0.335
ALB, mean (SD)	34.37 (4.57)	36.181 (4.02)	34.995 (4.46)	*t*-test	0.016
AST, median (P25, P75)	25 (20, 34)	26 (22, 33)	26 (21, 34)	Mann–Whitney *U* test	0.571
ALT, median (P25, P75)	15 (11, 24)	17 (11, 23)	15.6 (11, 24)	Mann–Whitney *U* test	0.986
ALP, median (P25, P75)	70 (60, 81)	71 (53, 83)	70.5 (60, 82)	Mann–Whitney *U* test	0.882
GGT, median (P25, P75)	23 (16, 38)	28 (16, 39)	24 (16, 39)	Mann–Whitney *U* test	0.596
LDH, median (P25, P75)	193 (166, 230)	212 (179, 256)	197.5 (168.5, 232)	Mann–Whitney *U* test	0.118
CK, median (P25, P75)	97 (67, 148)	99 (81, 166)	98.5 (71.25, 152)	Mann–Whitney *U* test	0.290
CK_MB, median (P25, P75)	15 (12, 18)	16 (12, 22)	15 (12, 20)	Mann–Whitney *U* test	0.073
IL-6, median (P25, P75)	10.40 (5.06, 23.60)	10.05 (6.49, 24.69)	10.23 (5.30, 24.24)	Mann–Whitney *U* test	0.481
IL-2, median (P25, P75)	1.29 (0.92, 2.04)	1.26 (0.8, 1.84)	1.27 (0.92, 1.89)	Mann–Whitney *U* test	0.347
IL-4, median (P25, P75)	1.49 (1.10, 1.94)	1.17 (0.91, 2.18)	1.435 (1.01, 2.01)	Mann–Whitney *U* test	0.348
IFN-γ, median (P25, P75)	2.71 (2.13, 3.56)	2.73 (2.01, 3.45)	2.71 (2.11, 3.45)	Mann–Whitney *U* test	0.822
TNF-α, median (P25, P75)	1.56 (1.10, 1.910)	1.45 (1.16, 1.93)	1.52 (1.14, 1.91)	Mann–Whitney *U* test	0.932
IL-10, median (P25, P75)	4.04 (2.92, 6.26)	4.54 (3.13, 10.8)	4.275 (2.97, 7.13)	Mann–Whitney *U* test	0.147

The median age of the cohort was 71 years (IQR: 62, 77), with no significant difference in age distribution between the HT and non-HT groups (*p* = 0.298). The male-to-female ratio was similar between the two groups (*p* = 0.923). The association between HT and interventional treatment methods was analyzed using chi-square tests. The distribution of treatment modalities—MT, balloon angioplasty combined with stenting, and hybrid treatment—did not show a statistically significant difference between the HT and non-HT groups (*p* = 0.663).

Other factors, such as hyperlipidemia, a history of cerebrovascular events, smoking, and alcohol consumption, showed no significant differences between the groups. Regarding vascular risk factors, a significantly higher proportion of patients with HT had DM (30.2% vs. 13.9%, *p* = 0.015) and AF (60.4% vs. 35.6%, *p* = 0.003).

The imaging findings revealed that the median ASPECTS score was comparable between the two groups, with no significant difference observed (HT group: 9 [IQR: 8, 10] vs. non-HT group: 10 [IQR: 8, 10], *p* = 0.295). The distribution of TOAST classifications differed significantly between the groups (*p* = 0.042), with a higher proportion of large artery atherosclerosis (TOAST_1) in the HT group (52.8% vs. 36.6%). Conversely, the proportion of cardioembolic stroke (TOAST_2) was lower in the HT group compared to the non-HT group (35.8% vs. 52.5%).

A significant difference was observed between the two groups in terms of the obstructed blood vessel variable (*p* = 0.010). Patients in the HT group were more likely to have occlusions in large proximal vessels, such as the ICA (obstructed blood vessel_1) (56.6% vs. 30.7%). However, only 3.8% of patients in the HT group had basilar artery occlusions (obstructed blood vessel_5) compared to 11.9% in the non-HT group.

Hemodynamic parameters also showed significant differences. The SBP and DBP were significantly higher in the HT group (SBP: 140 mmHg [IQR: 130, 150] vs. 130 mmHg [IQR: 126, 140], *p* = 0.003; DBP: 80 mmHg [IQR: 70, 83] vs. 70 mmHg [IQR: 70, 80], *p* < 0.001). Neurological severity, as assessed by the NIHSS score, was also significantly higher in the HT group (mean ± SD: 21.76 ± 5.78 vs. 18.77 ± 6.79, *p* = 0.007).

Laboratory findings revealed several significant differences between the groups. Patients in the HT group exhibited higher blood glucose levels (median: 7.88 mmol/L [IQR: 6.65, 9.29] vs. 7.02 mmol/L [IQR: 6.14, 8.07], *p* = 0.015), red blood cell counts (RBC) (4.24 ± 0.61 vs. 3.97 ± 0.57, *p* = 0.008), and serum albumin levels (mean ± SD: 36.18 ± 4.02 g/L vs. 34.37 ± 4.57 g/L, *p* = 0.016). However, other coagulation markers (D-dimer, PT, FIB, APTT) and inflammatory markers (IL-6, IL-10, CRP) did not show statistically significant differences between the HT and non-HT groups.

### 3.2 Selection of variables associated with HT by LASSO model

To identify independent predictors of HT following EVT, a two-step regression analysis was performed. Variables with *p*-values < 0.10 in the univariate analysis were first subjected to LASSO regression to reduce dimensionality and select relevant predictors. These predictors were subsequently included in a multivariate logistic regression model to determine independent risk factors for HT.

**Figure 1:**
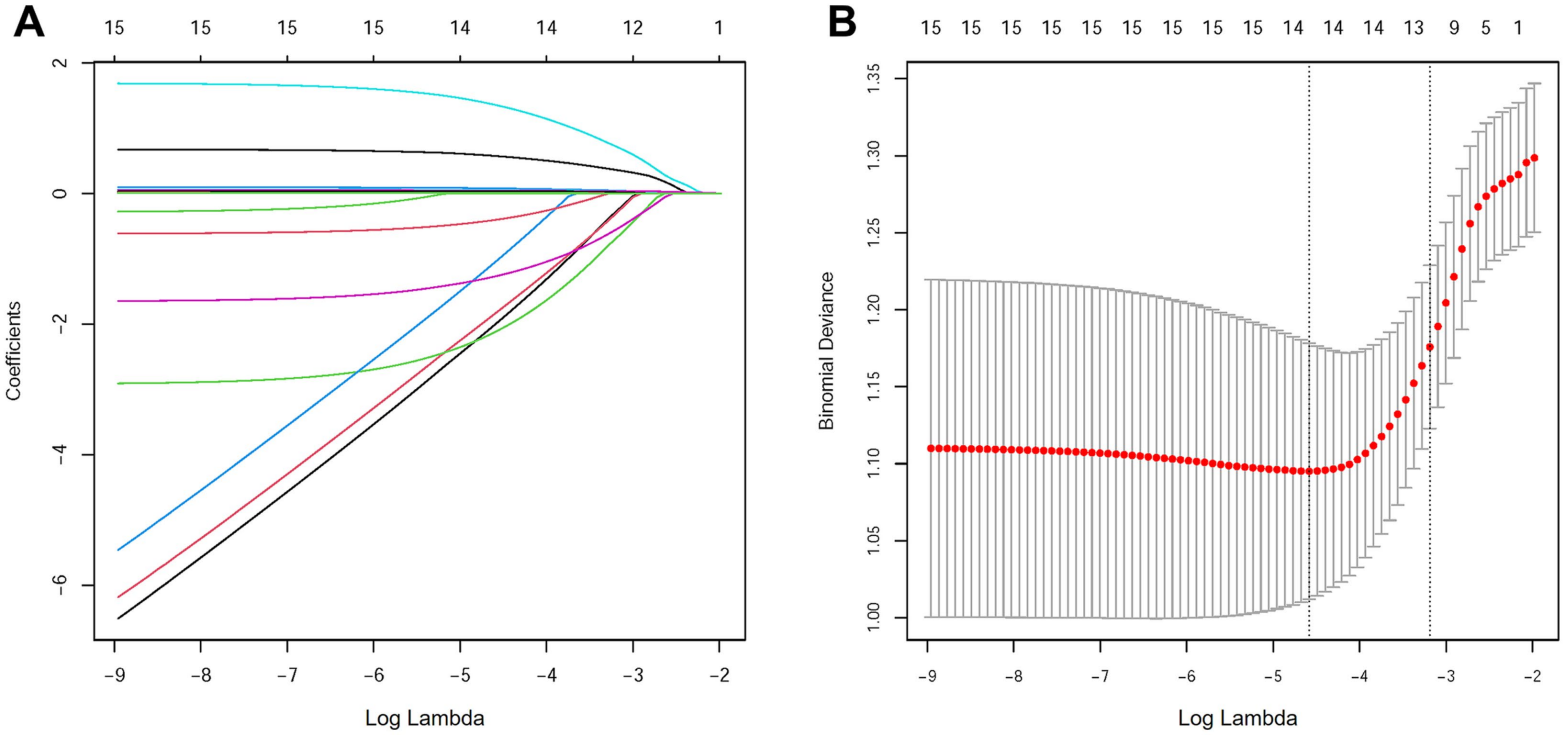
Selection of predictive variables using LASSO regression. **(A)** Characteristics of variable coefficient changes. This path diagram illustrates the evolution of the regression coefficients as the regularization parameter (*λ*) increases. **(B)** Determining the optimal value for parameter λ in the LASSO regression model using the cross-validation method.

LASSO regression was applied to the variables identified in the univariate analysis using cross-validation to select the optimal penalty parameter, *λ* (Figure 1). We selected lambda.1se which was 0.041 as the optimal *λ*. This step resulted in the selection of 12 predictors: DM, AF, related drug history, obstructed blood vessel, TOAST, SBP, DBP, NIHSS, RBC, GLU, ALB, CK_MB. The coefficient path diagram demonstrated the shrinkage of coefficients as λ increased, with the above variables remaining significant contributors to the model.

### 3.3 Multivariate logistic regression analysis HT risk

The variables selected through LASSO regression were further analyzed using multivariate logistic regression to identify independent predictors of HT. The logistic regression model identified six independent predictors of HT: Obstructed blood vessel_5 (basilar artery occlusion), TOAST_2 (cardioembolic stroke), AF, ALB, NIHSS score, and DBP. The results of the logistic regression analysis are presented in Table 2.

**Table 2:** Multivariate logistic regression analysis of factors associated with hemorrhagic transformation (HT) in acute ischemic stroke (AIS) patients.

Variable	Regression coefficient *β*	Standard error *S*	Wald value	OR	95% CI	*p*_value
Obstructed blood vessel_5	−2.516	1.013	−2.483	0.081	0.011–0.589	0.013
TOAST_2	−1.603	0.520	−3.083	0.201	0.073–0.558	0.002
Atrial fibrillation	1.817	0.514	3.536	6.152	2.247–16.845	0.0004
ALB	0.136	0.050	2.739	1.145	1.039–1.262	0.006
NIHSS	0.078	0.032	2.426	1.081	1.015–1.151	0.015
DBP	0.055	0.023	2.433	1.057	1.011–1.104	0.015

Multivariate logistic regression analysis demonstrated that AF (OR: 6.152; 95% CI: 2.247–16.845, *p* = 0.0004), ALB (OR: 1.145; 95% CI: 1.039–1.262, *p* = 0.006), NIHSS score (OR: 1.081; 95% CI: 1.015–1.151, *p* = 0.015), and DBP (OR: 1.057; 95% CI: 1.011–1.104, p = 0.015) were identified as independent risk factors for HT in AIS patients undergoing EVT.

In our analysis, basilar artery occlusion (Obstructed blood vessel_5) was found to be a protective factor for HT, with an OR of 0.081 (95% CI: 0.011–0.589, *p* = 0.013). Consequently, the other four types of vessel occlusion were identified as risk factors for HT. Similarly, the multivariate logistic regression analysis showed that TOAST_2 (cardioembolic stroke) was a protective factor for HT, with an OR of 0.201 (95% CI: 0.073–0.558, *p* = 0.002). In contrast, the other four subtypes of ischemic stroke according to the TOAST classification were identified as risk factors for HT.

### 3.4 Development and validation of the predictive model

#### 3.4.1 Construction of the nomogram model

**Figure 2:**
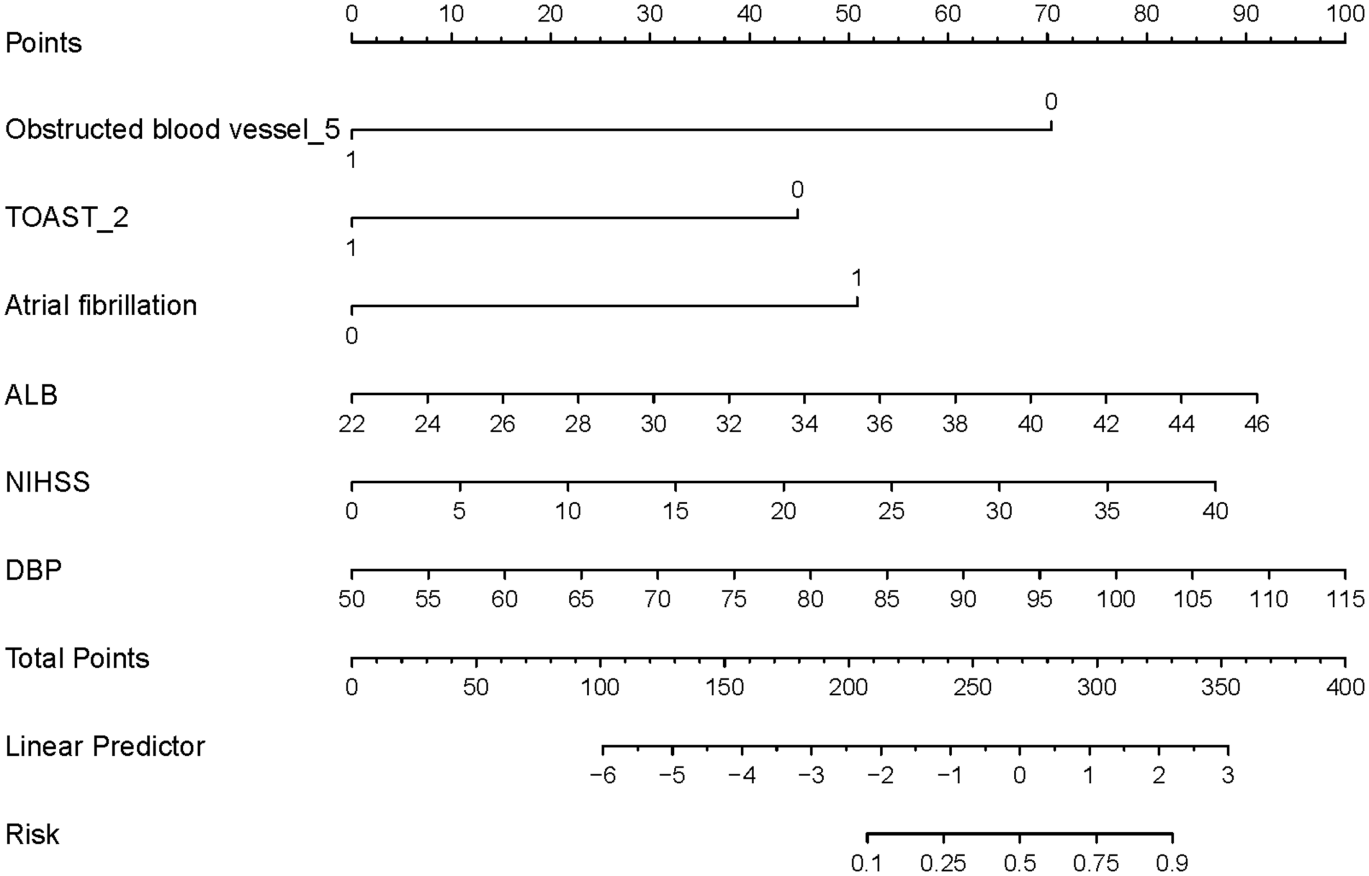
Nomogram for predicting HT in AIS patients undergoing EVT. The predictors include Obstructed blood vessel_5 (basilar artery occlusion), TOAST_2 (cardioembolic stroke), atrial fibrillation, albumin (ALB) levels, NIHSS score, and diastolic blood pressure (DBP). HT, hemorrhagic transformation; AIS, acute ischemic stroke; EVT, endovascular therapy.

Based on the independent predictors identified through multivariate logistic regression analysis, a nomogram was developed to estimate the probability of HT following EVT in patients with AIS (Figure 2). The predictors included in the model were: Obstructed blood vessel_5 (basilar artery occlusion), TOAST_2 (cardioembolic stroke), atrial fibrillation, albumin (ALB) levels, NIHSS score, and diastolic blood pressure (DBP).

The nomogram assigns a specific point value to each predictor based on its contribution to the risk of HT. For instance, higher NIHSS scores, elevated ALB levels, increased DBP, and the presence of atrial fibrillation contribute to a higher total score, indicating an elevated risk of HT. Conversely, if the obstructed blood vessel is not classified as category 5 (basilar artery occlusion), or if the stroke subtype is not TOAST_2 (cardioembolic stroke), the risk of HT increases.

Each predictor is scored individually, and the total score is calculated by summing the points for all variables. This total score is then mapped to a linear predictor and converted into a probability of HT using the risk scale located at the bottom of the nomogram. This approach provides clinicians with an intuitive tool to assess individualized HT risk, facilitating personalized risk stratification and decision-making.

The nomogram model integrates both clinical and biochemical parameters, reflecting the multifactorial nature of HT development. Its graphical representation allows clinicians to efficiently evaluate risk by aligning a patient’s clinical and laboratory data with the corresponding points and calculating the total score. This user-friendly design enhances its applicability in clinical practice and supports tailored treatment.

#### 3.4.2 Model performance evaluation

The performance of the nomogram was evaluated using three key metrics: the area under the receiver operating characteristic curve (AUC-ROC), calibration curves, and decision curve analysis (DCA).

**Figure 3:**
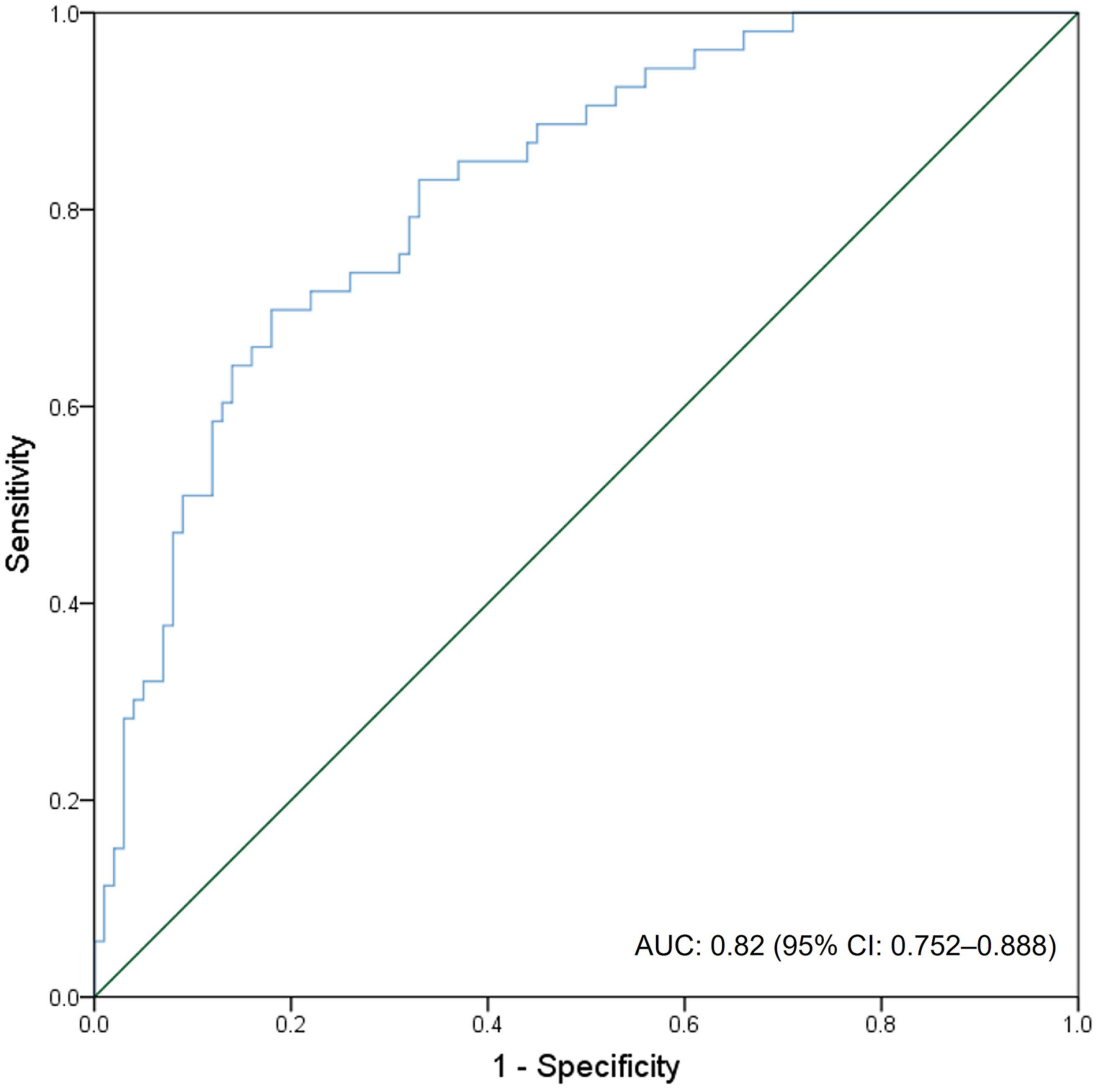
ROC curve and AUC of the nomogram for predicting HT in AIS patients undergoing EVT. ROC, receiver operating characteristic; AUC, area under the ROC curve; HT, hemorrhagic transformation; AIS, acute ischemic stroke; EVT, endovascular therapy.

The model exhibited excellent discriminatory ability (Figure 3), achieving an AUC-ROC value of 0.82 (95% CI: 0.752–0.888). This indicates that the nomogram has a strong capacity to differentiate between patients with and without hemorrhagic transformation (HT). The sensitivity and specificity of the model were 64.2 and 86.1%, respectively.

**Figure 4:**
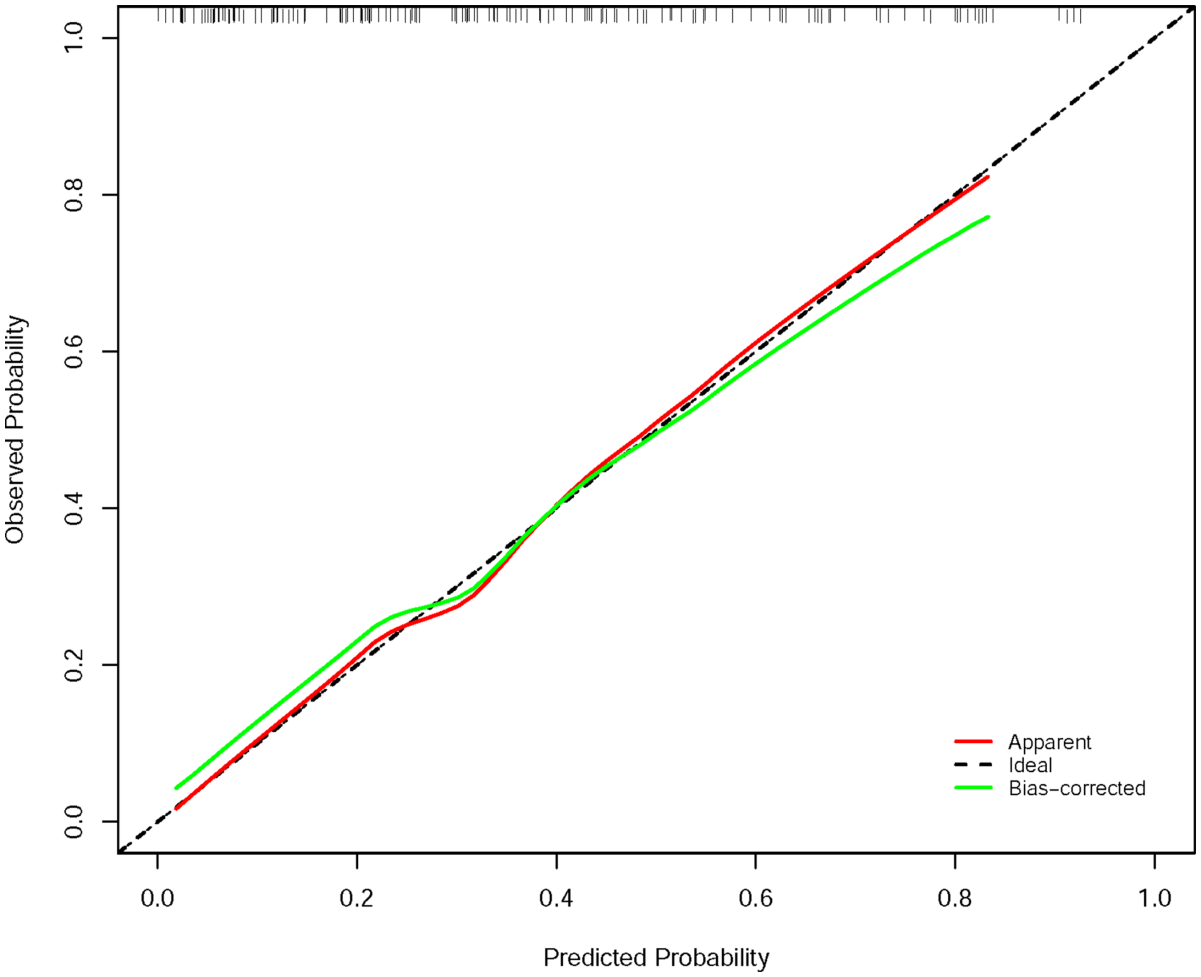
Calibration curve for evaluating the agreement between the nomogram predicted probability and the actual probability.

The calibration curve (Figure 4) demonstrated that the predicted probabilities of HT closely matched the observed probabilities. This finding highlights that the model is well-calibrated and capable of providing accurate probability estimates for HT risk.

**Figure 5:**
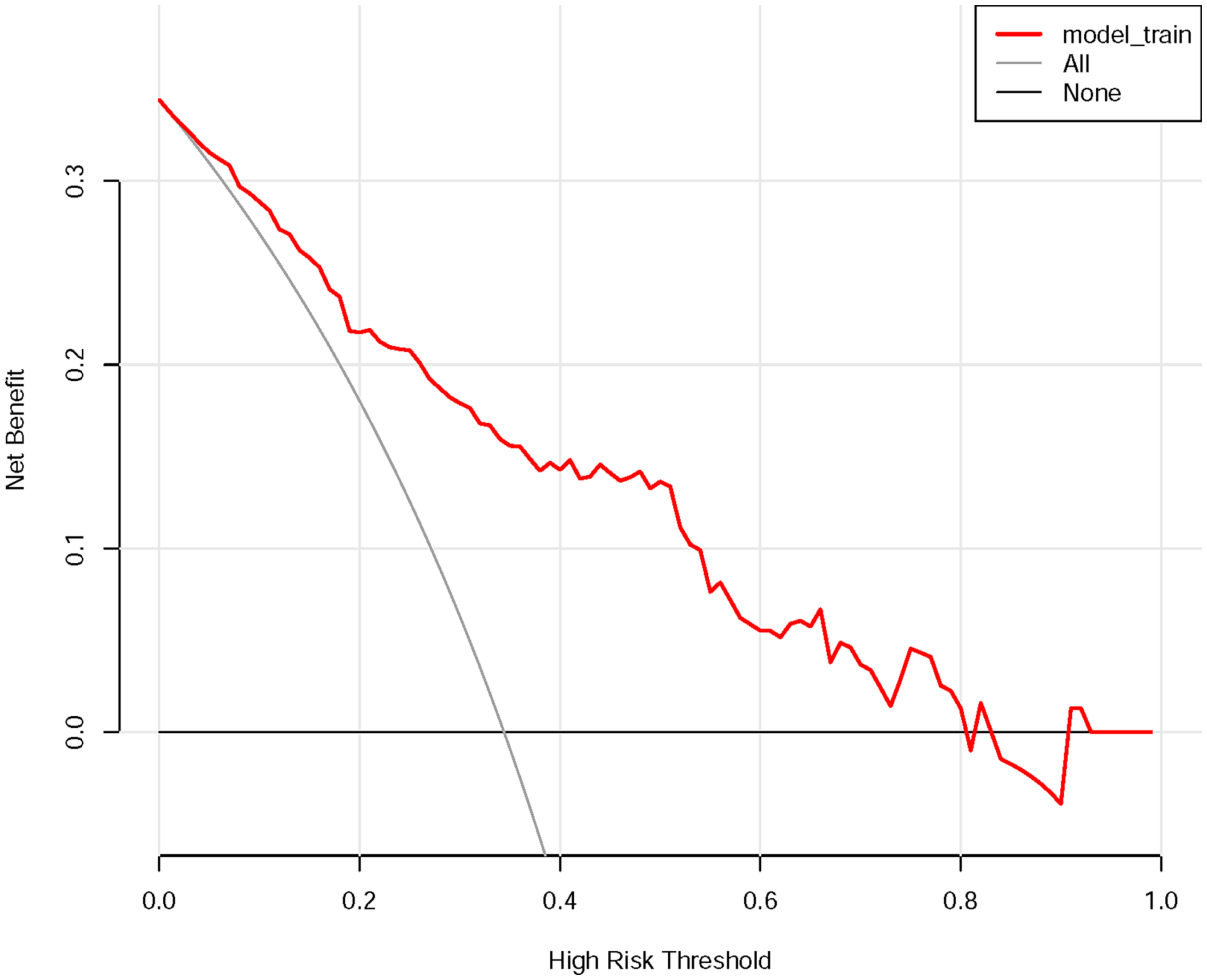
DCA of the nomogram for evaluating the clinical applicability of the model. DCA, decision curve analysis.

The DCA (Figure 5) further illustrated the clinical utility of the nomogram across a range of threshold probabilities. The DCA indicated that predicting HT risk with this nomogram provided a net clinical benefit when the threshold probability of HT was below 0.8.

In summary, the nomogram demonstrated robust discriminatory power, strong calibration, and notable clinical utility, as evidenced by the results of the DCA.

## 4 Discussion

In this study, we developed and validated a novel nomogram-based predictive model to assess the risk of HT in patients with AIS undergoing EVT. Through multivariate logistic regression analysis, six independent predictors were identified: AF, ALB, baseline NIHSS score, DBP, cardioembolic stroke classification (TOAST_2), and basilar artery occlusion (Obstructed blood vessel_5). The nomogram demonstrated excellent predictive performance, achieving an AUC-ROC of 0.82, indicative of strong discriminatory ability. Calibration analyses showed a high degree of agreement between predicted and observed probabilities, while DCA indicated potential clinical utility by assessing the net benefit of using the nomogram within this development cohort. It is crucial to emphasize, however, that while DCA is a valuable tool for evaluating the potential practical value of a predictive model under various decision thresholds, it does not constitute comprehensive clinical validation. True clinical validation of this nomogram as a decision-making aid would necessitate prospective evaluation in diverse, external patient populations to confirm its real-world performance, impact on clinical decisions, and ultimately, patient outcomes. This prospective validation remains a critical next step, as acknowledged in the limitations of our current study.

The results of this study underscore the multifactorial nature of HT risk, highlighting the importance of incorporating clinical, laboratory, and imaging variables into predictive frameworks. The inclusion of both protective factors, such as basilar artery occlusion and TOAST_2, and risk factors, such as AF, ALB, and NIHSS score, emphasizes the nuanced interactions between these variables in determining HT outcomes. These findings provide clinicians with a practical and intuitive tool for estimating HT risk, enabling early identification of high-risk patients and optimizing perioperative management strategies.

A crucial aspect of this predictive model is its practical application in guiding clinical decision-making. It is imperative to state that this nomogram is not intended to serve as a sole determinant for withholding EVT. The decision to perform EVT is complex, primarily guided by established clinical and imaging criteria indicating potential for good functional outcome. Rather, this tool is designed to be adjunctive, enhancing the clinician’s ability to stratify HT risk. For patients identified by the nomogram as having a high probability of HT, clinicians might be prompted to implement more intensive peri-procedural monitoring, such as stricter blood pressure control within target ranges, careful consideration of post-procedural antithrombotic regimens, heightened neurological surveillance, and potentially earlier or more frequent follow-up imaging to detect and manage HT promptly. The decision curve analysis supports the nomogram’s clinical utility when the threshold probability is < 0.8, indicating its value in risk assessment; however, this does not translate to a specific threshold for denying EVT. The establishment of a definitive risk–benefit threshold for EVT based on HT risk alone would require extensive prospective validation and consideration of multiple outcome factors. The current model’s strength lies in its ability to provide individualized risk probabilities, thereby facilitating more informed discussions with patients and their families regarding procedural risks and contributing to tailored peri-operative care planning aimed at mitigating such risks.

The findings of this study align with prior research that has identified critical risk factors for HT, such as AF, NIHSS score, and elevated ALB levels, while also offering new insights into the protective roles of specific vascular and stroke subtype characteristics. The NIHSS score is a well-established indicator of stroke severity and infarct size, both of which are closely associated with HT risk. A higher NIHSS score typically reflects a larger area of ischemic brain tissue, which undergoes more profound and widespread BBB breakdown. This extensive BBB damage involves the degradation of tight junction proteins, basal lamina components, and increased activity of matrix metalloproteinases (MMPs), creating a highly permeable vascular bed (17). Previous studies, such as Huang et al., identified baseline NIHSS score, admission serum glucose, and fibrinogen as independent predictors of HT following intravenous thrombolysis (18). Similarly, Liu et al. demonstrated that pre-thrombolytic NIHSS score and glucose levels were significant risk factors for both symptomatic and non-symptomatic HT (19). This study corroborates the importance of the NIHSS score as a robust predictor of HT across different therapeutic modalities, including EVT. However, while glucose has frequently been identified as a key predictor in thrombolytic cohorts, it was not included in our final model, possibly reflecting differences in the pathophysiology of HT between thrombolysis and EVT populations.

The AF is often associated with larger infarct volumes and an increased risk of HT due to the embolic burden and the use of anticoagulation therapy (20). A meta-analysis identifying risk factors for HT reported that AF and NIHSS score are common predictors of any intracerebral hemorrhage (ICH) following reperfusion therapies, IVT and EVT (21). Additionally, a multicenter prospective clinical trial demonstrated that AF was significantly associated with an increased risk of any type of ICH after mechanical thrombectomy (MT) (OR: 2.198; 95% CI: 1.099–4.395; *p* = 0.026). Notably, the impact of AF on ICH risk was partially attributed to adjusted anticoagulation status and an increased number of thrombectomy attempts (22). Zubair and Sheth (23) and Yaşar et al. (24) also highlighted the association between AF and poor outcomes following stroke, further supporting its inclusion in our model. These findings underscore the critical need for careful anticoagulation management and close monitoring of AF patients undergoing EVT. The pro-hemorrhagic influence of AF in the context of EVT may stem from several physiological underpinnings. AF is often associated with the generation of larger cardiac emboli, which can occlude more proximal cerebral vessels, leading to larger infarct volumes. Such extensive ischemic tissue is characterized by severe disruption of the BBB integrity, making it highly susceptible to hemorrhage upon reperfusion (25). Furthermore, chronic AF may induce a systemic pro-inflammatory state and endothelial dysfunction, which could further compromise cerebrovascular resilience to the mechanical and hemodynamic stresses of thrombectomy and reperfusion (26).

Our study identified a positive association between higher ALB levels and HT, which contrasts with previous reports. For instance, Wu et al. reported no significant difference in ALB levels between HT and non-HT patients following EVT (8). Other studies have emphasized the neuroprotective effects of albumin, citing its antioxidative, antiapoptotic, and anti-inflammatory properties. Several studies have also shown that lower albumin levels are associated with higher rates of post-stroke hemorrhage, potentially due to increased vascular permeability and inflammation in patients with hypoalbuminemia (27, 28). The discrepancies between these findings may stem from differences in baseline patient characteristics and therapeutic approaches. The inclusion of ALB as an independent predictor in our model highlights the growing recognition of systemic factors, such as albumin, in influencing HT outcomes.

It is important to acknowledge that while albumin levels were identified as an independent predictor, the immediate availability of this and other laboratory markers at the precise moment of EVT decision-making can vary. However, these markers are often part of the initial comprehensive stroke workup, with results typically available in the early phase of patient management. Thus, the nomogram’s utility extends beyond the go/no-go EVT decision to encompass early risk stratification, which can inform peri-procedural vigilance and management strategies for patients undergoing or scheduled for EVT.

Elevated blood pressure, particularly DBP, was also identified as a contributing factor to HT in our study. Elevated blood pressure reflects increased hemodynamic stress, which can exacerbate reperfusion injury and compromise the integrity of the blood–brain barrier (29). A large-scale survey conducted in the United States reported that >60% of stroke patients presented with elevated blood pressure (30). In a study investigating EVT for stroke, the proportion of patients with a history of hypertension was significantly higher among those who developed symptomatic ICH compared to those with non-symptomatic ICH, 79.4% vs. 58.8% (31). Another study analyzing risk factors and predictors of early HT after reperfusion therapy (including IVT and MT) identified baseline high blood pressure as an independent risk factor for early HT (32). Liu et al. also emphasized the importance of blood pressure control in reducing HT risk after thrombolysis (19). However, previous studies have not separately analyzed SBP and DBP to characterize their potentially distinct roles in HT. Our findings suggest that DBP, rather than SBP, serves as a predictor of HT following EVT. These results highlight the importance of maintaining stable blood pressure for preventing HT after successful EVT and emphasize the necessity of strict perioperative blood pressure management.

The use of radiomics in predicting HT has been explored in studies such as Heo et al. (33), which demonstrated that radiomic features extracted from non-contrast CT scans could provide highly accurate HT predictions, with an AUC-ROC of 0.986. Previous studies, including Zubair and Sheth (23), identified large vessel occlusion (TOAST_1) and cardioembolic stroke (TOAST_2) as risk factors for HT, potentially due to their association with larger infarct sizes and higher embolic burden. However, our study diverges from these findings, identifying TOAST_2 (cardioembolic stroke) and basilar artery occlusion as protective factors against HT.

This suggests that other types within the TOAST and Obstructed Blood Vessel classifications can be considered risk factors for HT. For example, Obstructed blood vessel_1 (ICA): a clinical trial demonstrated that ICA occlusion is an independent risk factor for HT within 24 h after thrombectomy in AIS patients (34). A meta-analysis including 1,903 patients with large artery atherosclerosis (TOAST_1, LAA) and 3,214 patients with cardioembolism (TOAST_2, CE) found no significant difference in symptomatic intracerebral hemorrhage (sICH) rates between LAA and CE patients (OR: 1.09; 95% CI: 0.71–1.66). However, LAA patients had a higher mortality risk (OR: 1.44; 95% CI: 1.24–1.71) (35). The specific causes or mechanisms underlying these differences remain unclear, but we infer that they may be related to variations in study populations, treatment protocols, or statistical methodologies, underscoring the need for further investigation to validate and contextualize these findings. These results also emphasize the importance of tailoring HT risk assessments to specific stroke subtypes and vascular territories.

It is also worth noting that prior research has demonstrated the significant role of inflammation in HT (36). For instance, in AIS patients undergoing EVT, a higher admission neutrophil-to-lymphocyte ratio (NLR) in peripheral blood was independently associated with ICH risk (OR: 1.09; 95% CI: 1.00–1.20; *p* = 0.040) (37). Pro-inflammatory factors secreted by neutrophils, such as TNF-*α* and IL-6, promote the expression of MMP-9, which exacerbates endothelial damage in cerebral vessels following stroke and increases the risk of hemorrhagic complications (38). However, in our study, these inflammatory markers did not demonstrate an independent association with HT. This discrepancy may be attributed to differences in the timing of biomarker measurements, sample sizes, or study populations.

This study has several limitations. First, it was conducted at a single center, which may limit the generalizability of the findings to other populations or healthcare settings. Differences in patient demographics, treatment protocols, and resource availability could influence the applicability of the nomogram in broader clinical contexts. Multicenter studies are needed to validate the model in diverse populations. Second, the predictors included in this study were measured at baseline, and the dynamic changes of these factors during the perioperative period were not accounted for. For example, fluctuations in blood pressure or inflammatory markers during and after EVT could provide additional insights into HT risk. Future studies should explore the incorporation of dynamic predictors to improve the model’s predictive accuracy. Third, although the sample size was adequate for the primary analysis, subgroup analyses based on specific EVT modalities or patient characteristics (e.g., age, sex) were limited by statistical power. Larger datasets are needed to explore potential interactions and refine the model’s predictive performance in specific subgroups. Fourth, the primary endpoint for this nomogram was the occurrence of any radiological HT. While this is a clinically relevant outcome, future studies should aim to develop models specifically predicting more severe HT subtypes (e.g., parenchymal hematomas PH1/PH2 according to ECASS criteria) or symptomatic HT, as these have more direct and severe prognostic implications. Analyzing predictors for these distinct endpoints could offer more refined risk stratification for guiding clinical decisions. In conclusion, while this study provides a robust and clinically meaningful predictive model for HT risk after EVT, its limitations underscore the need for further prospective, multicenter studies to validate and refine the findings.

## 5 Conclusion

This study developed and validated a novel nomogram that integrates six independent predictors: Obstructed blood vessel, TOAST classification, AF, ALB, NIHSS score, and DBP. The nomogram demonstrates robust predictive performance, strong calibration, and notable clinical utility, making it a valuable tool for estimating the risk of HT in AIS patients undergoing EVT. By providing accurate, individualized risk assessments, this model has the potential to improve clinical outcomes and mitigate the burden of hemorrhagic complications in stroke care.

## Data Availability

The original contributions presented in the study are included in the article/supplementary material, further inquiries can be directed to the corresponding author.
